# The complete chloroplast genome of *Dunnia sinensis* (Rubiaceae): a monotypic species endemic to Guangdong, China

**DOI:** 10.1080/23802359.2020.1715876

**Published:** 2020-01-24

**Authors:** Ying Zhang, Sheng Chen, Xianyi Xu, Ruijiang Wang

**Affiliations:** a Key Laboratory of Plant Resources Conservation and Sustainable Utilization, South China Botanical Garden, Chinese Academy of Sciences, Guangzhou, China;; b College of Life Sciences, University of Chinese Academy of Sciences, Beijing, China;; c College of Life Sciences, South China Agricultural University, Guangzhou, China

**Keywords:** *Dunnia*, plastome, Rubiaceae, phylogeny

## Abstract

*Dunnia sinensis*, a monotypic genus of the Rubiaceae, is an Endangered species endemic to China. Its complete chloroplast genome was determined to be 154,909 bp in length and the GC content was 37.80%. The sequence includes a large single-copy region of 84,894 bp, a small single-copy region of 16,973 bp, and the inverted region of 26,521 bp in length. It contains 130 genes, including 84 protein-coding genes, 37 tRNA genes, and 8 rRNA genes. The ML and BI analyses revealed *D. sinensis* was closely related to *Galium mollugo* and *Leptodermis scabrida* with strong bootstrap values belonging to the subfamily Rubioideae.

*Dunnia sinensis* Tutch., a monotypic genus of Rubiaceae, is endemic species distributed in the south of Guangdong Province in China (Tutcher 1905; Ridsdale 1978). It is a woody shrub with a height of up to 3 m, flowering from late April to May and fruiting in October and November. Besides great ornamental value, *Dunnia sinensis* was used as an anti-inflammatory drug in folk medicine (Wei et al. 2000). Over the past decades, its habitats, usually along streams or hillside at 100–600 m of altitude, have been seriously destructed for economic development, and therefore it was listed as the national key protected wild species (Chiang et al. 2002; Ge et al. 2002; Wang and Xie 2004). Although its phylogeographic pattern and phylogenetic studies were conducted previously (Chiang et al. 2002; Ge et al. 2002; Rydin et al. 2008), no genome has been reported until now. Therefore, we reported the complete chloroplast (cp) genome of this endangered endemic species (GenBank Accession Number: MN883829) to provide useful genomic information for evolutionary dynamics and conservation evaluation.

The fresh and healthy leaves of *D. sinensis* were collected from Taishan County, Jiangmen City, Guangdong Province of China (22°14′13.57″N, 112°56′.50″E, 66 m). The voucher specimen (*Ruijiang Wang*, *Gangtao Wang*, *Ying Zhang 4395*) was deposited in the South China Botanical Garden Herbarium, Chinese Academy of Sciences (IBSC). The total genomic DNA was extracted from the silica-gel dried leaves following the modified CTAB method (Doyle and Doyle 1987). Then, the genomic library (paired-end, PE = 150 bp) was sequenced on an Illumina Hiseq X Ten platform at Beijing Genomics Institute (Shenzhen, China). Totally, 2 Gb sequence reads were obtained and used to assemble the cp genome after filtering and trimming the low-quality reads and adaptor sequences. The complete cp genome assembly was executed on NOVOPlasty 2.6.3 (Dierckxsens et al. 2017) with the default k-mer of 39–59, while Geneious version 11.0.3 (Kearse et al. 2012) was used to annotate the genome. *Galium mollugo* (GenBank accession number: NC_036970) was used as the reference plastid genome for assembling and annotation. The tRNA genes were annotated on ARAGORN (Laslett and Canbäck 2004). 

The complete cp genome of *D. sinensis* was 154,909 bp with the typical quadripartite structure of angiosperms, including a small single-copy region (SSC) of 16,973 bp, a large single-copy region (LSC) of 84,894 bp, and the inverted repeat region (IR) of 26,521 bp. The genome harbored 130 genes, including 84 protein-coding genes, 37 tRNA genes, and 8 rRNA genes. The overall GC content in the cp genome of *D. sinensis* was 37.80%, in which the corresponding value of the SSC, LSC, and IR region was 32.10, 35.70, and 42.90%, respectively.

The cp genome sequences of 13 species (Zhang et al. 2020) within Rubiaceae were used to infer the phylogenetic position of *D. sinensis*, using *Buddleja colvilei* Hook. f. & Thoms (Loganiaceae) (NC_042766) as outgroup. A Maximum Likelihood (ML) tree was constructed by using IQ-TREE (Nguyen et al. 2015) with 1000 bootstrap replicates under the GTR + F+R4 substitution model. Bayesian inference (BI) was also performed with MrBayes v.3.2.6 (Ronquist et al. 2012). The ML and BI trees were consistent and robust, showing *D. sinensis* had a close relationship with *Galium mollugo* and *Leptodermis scabrida*, which formed an independent clade of the subfamily Rubioideae (Figure 1). The phylogenetic relationship of *D. sinensis* with genomic data was uncovered for the first time, largely enriching genetic resources for resolving the complex phylogeny relationship of Rubiaceae.

**Figure 1.**
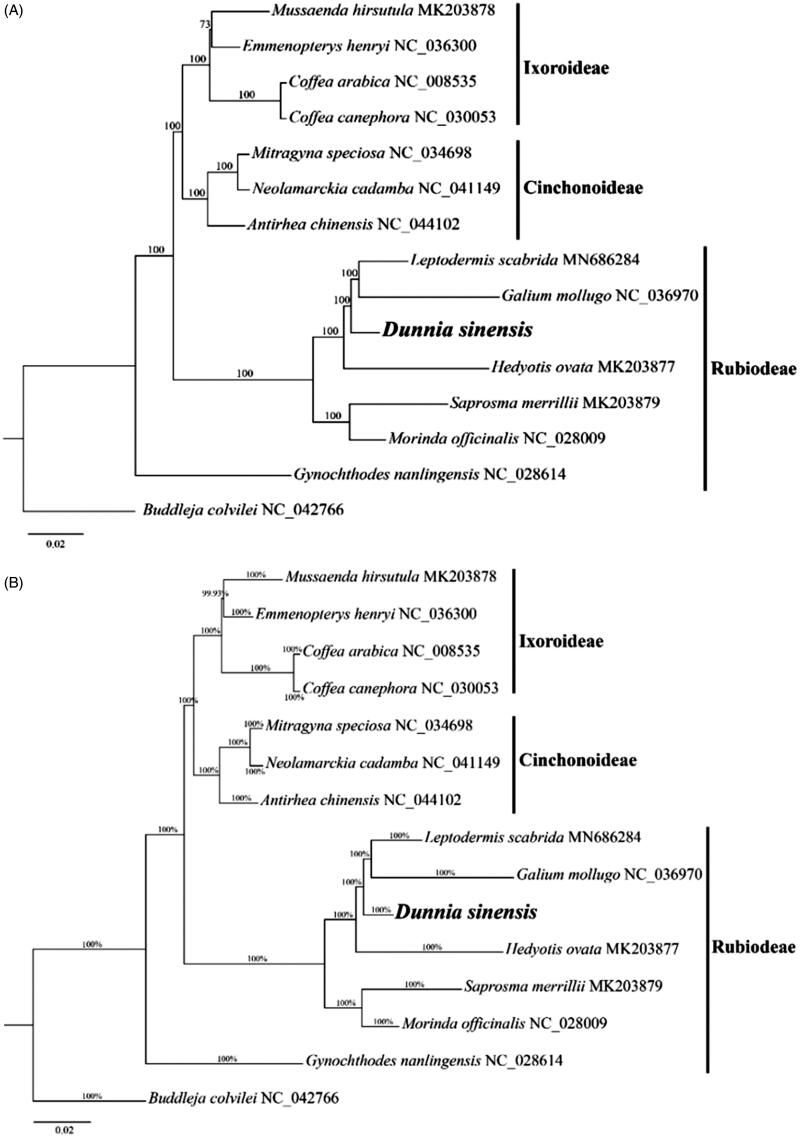
Maximum likelihood tree (A) and Bayesian inference (B) based on 14 (including *Dunnia sinensis*) whole cp genome sequences in Rubiaceae. Bootstrap support values were shown at the branches. *Buddleja colvilei* served as outgroup.
